# The Operative Treatment of Inguinal Hernia in Hospital

**Published:** 1917-04-07

**Authors:** Arthur A. Straton

**Affiliations:** Temp. Capt. R.A.M.C.


### THE OPERATIVE TREATMENT OF INGUINAL HERNIA IN HOSPITAL.

By ARTHUR A. STRATON, M.D. Lond., F.R.C.S. Edin. (Temp. Capt. R.A.M.C.)

(*Continued from March* 31, *page* 516.)

Where Kocher's operation with reinforcement has been performed—*i.e.* where the weak spot in the peritoneum has been fixed to the abdominal wall in a new position, and the stretched muscles have been pleated or strengthened—the patient may be allowed to lie upon either side from the first, and to sit, or even to kneel, for purposes of micturition as early as the second day. The stitches or clips should be removed upon the eighth day, unless healing by first intention should not have occurred, and he will from that time be allowed to go to the lavatory whenever necessary. He will be allowed to dress himself and to sit about the ward upon the ninth day, and to return home upon the eleventh day without any undue risk of recurrence. He should report himself at the end of another month for examination, and should then be able to resume any but the most laborious of occupations, which should not be attacked until after a further examination at the end of one more month.

Where the Bassini or Halstead operation has been performed, a longer rest in bed is probably necessary, and, in the majority of cases, the patient should not leave his bed much before the fifteenth day, and heavy work must be postponed until a much later date.

Infants may be sent home the night after the operation or upon the following day, provided that the home is clean, and that either a nurse is available for visiting the patient or the hospital is near enough to enable the child to be brought up for inspection every other day. This procedure will allow of greater freedom in the children's ward, often the spot of greatest congestion in the hospital: its results have proved satisfactory in many large hospitals.

### Conclusion.

The above remarks doubtless in some respects do not reflect the orthodox teaching, but at the same time they are put forward as the fruits of an experience of some years spent as resident in various hospitals, which has rendered possible a comparison between many different methods and routines in dealing with these cases. Each case must, of course, be judged upon its own merits, but there are certain points to be borne in mind with regard to the three main principles involved—namely, economy of time and labour, economy of beds, and economy of dressings, and these are as follows:

Firstly: That shaving may be done at home by the patient himself in many cases, whilst for the remainder a barber should be employed by the hospital to perform this duty. It is unfair to expect an over-worked house-surgeon, after a strenuous day spent in the operating theatre or in the casualty and out-patient departments, to utilise his spare time at night in shaving pubic hair; rather let it be done upon the table, as with emergency cases, in which iodine covers an abundance of unpreparedness.

Secondly: That wet compresses and indiscriminate enemata are unnecessary and harmful. Let the disbeliever experiment upon himself and be convinced.

Thirdly: That a night's rest before and after the operation is of greater value to a nervous patient than any amount of preparation. It is better to secure this with morphia than to allow a patient to lose ground from restlessness and worry.

Fourthly: That with treatment by the "reinforced Kocher" method, patients can with impunity leave hospital upon the eleventh day after operation, provided that healing has taken place by first intention.

Fifthly: That a thin film of wool soaked in collodion forms an ample dressing of itself, without pad and bandage, except in rare instances. It is clean, aseptic, and allows of a clear view of the field of operation without disturbance of the patient. Further, it represents an enormous economy of dressings in a year's work.

Sixthly: That a certain definite proportion of beds should be always retained for cases of emergency, and the "waiting-list" diminished by never allowing any of the remaining beds to lie empty for a single unnecessary day. Such I conceive to comprise a model routine for the treatment of hernia patients in hospital.

